# Neglected Patients with a Neglected Disease? A Qualitative Study of Lymphatic Filariasis

**DOI:** 10.1371/journal.pntd.0000128

**Published:** 2007-11-21

**Authors:** Myrtle Perera, Margaret Whitehead, David Molyneux, Mirani Weerasooriya, Godfrey Gunatilleke

**Affiliations:** 1 Marga Institute, Colombo, Sri Lanka; 2 Division of Public Health, University of Liverpool, Liverpool, United Kingdom; 3 Lymphatic Filariasis Support Centre, Liverpool School of Tropical Medicine, Pembroke Place, Liverpool, United Kingdom; 4 Filariasis Research, Training and Service Unit, Department of Parasitology, Faculty of Medicine, University of Ruhuna, Galle, Sri Lanka; Swiss Tropical Institute, Switzerland

## Abstract

**Background:**

Lymphatic filariasis (LF) is a so-called neglected tropical disease, currently overshadowed by higher-profile efforts to address malaria, tuberculosis, and HIV/AIDS. Despite recent successes in arresting transmission, some 40 million people who already have the disease have been largely neglected. This study aims to increase understanding of how this vulnerable, neglected group can be helped.

**Methods:**

We used purposive sampling to select 60 men and women with filarial lymphoedema (45 with filarial elephantiasis and 15 men with filarial hydrocoele) from the south of Sri Lanka in 2004–2005. Participants were selected to give a balance of men and women and poor and nonpoor, and a range of stages of the disease. Participants' experiences and the consequences of their disease for the household were explored with in-depth qualitative, semistructured interviews.

**Findings:**

LF was extremely debilitating to participants over long periods of time. The stigma attached to the condition caused social isolation and emotional distress, and delayed diagnosis and treatment, resulting in undue advancement of the disease. Free treatment services at government clinics were avoided because the participants' condition would be identifiable in public. Loss of income due to the condition was reported by all households in the sample, not just the poorest. Households that were already on low incomes were pushed into near destitution, from which it was almost impossible to escape. Affected members of low-income households also had less opportunity to obtain appropriate treatment from distant clinics, and had living and working conditions that made hygiene and compliance difficult.

**Significance:**

This highly vulnerable category of patients has low visibility, thus becoming marginalized and forgotten. With an estimated 300,000 total cases of elephantiasis and/or oedema in Sri Lanka, and around 300,000 men with filarial hydrocoele, the affected households will need help and support for many years to come. These individuals should be specially targeted for identification, outreach, and care. The global strategy for elimination is aimed at the cessation of transmission, but there will remain some 40 million individuals with clinical manifestations whose needs and problems are illustrated in this study.

## Author Summary

Lymphatic filariasis (LF) is a tropical disease causing extreme swelling of the limbs and male genitals. Despite recent successes in preventing transmission of the disease, some 40 million people worldwide who already have the disease have been largely neglected. We aimed to increase understanding of how this vulnerable, neglected group can be helped, by asking people with LF in Sri Lanka to recount their own experiences.

Study participants reported that LF was extremely debilitating over a long period of time. The social isolation from stigma caused emotional distress and delayed diagnosis and treatment. Free treatment services at government clinics were avoided because the participants' condition would be identifiable in public. Loss of income due to the condition was reported by all households, not only those of the poorest. Households that were already on low incomes were pushed into near destitution by LF. Low-income households also had fewer opportunities to obtain effective treatment from distant clinics, and had living and working conditions that made treatment more difficult.

With an estimated 300,000 people with swelling of the limbs in Sri Lanka, and around 300,000 men with swelling of the genitals, we conclude that the affected households will need help and support for many years to come, and offer suggestions for immediate action.

## Introduction

Recently, the profile of the “neglected diseases” [1],[2] has been enhanced by a renewed interest by policymakers, including the new Director-General of the World Health Organization (WHO). These diseases cause long-term morbidity, rather than high mortality, but have been overshadowed by higher-profile efforts to address malaria, tuberculosis, and HIV/AIDS [2]. Recent studies show extensive and underestimated morbidity for the neglected diseases [3], totalling around 56 million cumulative disability-adjusted life years, which is more than for malaria and tuberculosis [4]. Lymphatic filariasis (LF) is one of these diseases and one of the leading causes of disability, infecting some 120 million individuals, with a further 1.3 billion people at risk [5].

Some of the best “global health buys,” in terms of cost per disability-adjusted life years averted, are preventive chemotherapy for the control of intestinal helminths, elimination of LF, and control of onchocerciasis (the latter two programmes are based on drug donations) [6]. Treatment costs of such chemotherapy packages range from US$0.03 to US$1 [7]–[9], and it is recognised that cost savings by integration of NTD programmes can reach as much as 47% [10]. Economic rates of return on controlling the neglected diseases are 15%–30% [1].

The Global Programme to Eliminate Lymphatic Filariasis is arguably the most rapidly expanding global health intervention [5]. Since 2000, when nearly 12 million people were treated, the latest WHO figures show that around 381 million people received treatment in 2005 in 42 countries [5]. There is strong evidence to suggest that the WHO strategy has eliminated the transmission by mosquitoes of the causative agent *Wuchereria bancrofti* in Egypt [11],[12], whilst in other settings, including China, the disease is reported to have been eliminated [13] or transmission arrested [14].

These successes give an incomplete picture, because some 40 million people who are deformed, stigmatised, and disabled by the disease have been largely neglected. There are, however, promising interventions that could improve the quality of life and reduce the level of disability of patients. If effective interventions are to be successfully implemented, a greater understanding is required of the consequences of the disease for individuals and their families, the barriers they face to accessing the care they need, and their coping strategies. Whilst there have been studies in a number of countries on the social and cultural aspects of LF prior to the advent of the Global Programme to Eliminate Lymphatic Filariasis [15]–[17], we report here a recent in-depth study into the social and economic impact of filarial elephantiasis in Sri Lanka from the perspective of the people suffering from the disease themselves. The objective of the study was to inform future interventions and policy to help these vulnerable, neglected people. By doing so, it responds to needs for specific research identified in the most recent review of the sociocultural aspects of filariasis [18].

## Methods

### Setting and study population

Reference to LF in Sri Lanka has been traced to the 13th century AD [19]. Brugian filariasis, caused by *Brugia malayi*, was eliminated by chemotherapy and vector control through the Anti-Filariasis campaign, which began in 1947. The infection currently endemic in the country is due to *W. bancrofti*, and is presently confined to eight districts in the Southern, Western, and Northwestern Provinces [20]. Our study took place in three villages in Matara and one in Galle in 2004–2005. Further details of the geography, ecology, and social structure of the communities can be found in earlier published work [21].

For the qualitative study, systematic purposive sampling was used to select 60 participants with LF for in-depth interviews concerning their experiences and consequences of the disease. Participants were selected by poverty status, sex, and lymphoedema stage (Table 1).

**Table 1:** Sample Distribution—Interviewees by Sex, Lymphoedema Grade, and Income Category.

Stage of Lymphoedema	Description^a^	Household Income						Total
		Low Income		Middle Income		High Income		
		Male	Female	Male	Female	Male	Female	
1	Swelling is reversible overnight	—	1	—	—	—	2	3
2	Swelling does not resolve overnight or without active management	2	7	2	8	—	4	23
3	Swelling and presence of shallow skin folds	1	2	3	4	—	1	11
4	Presence of knobs, bumps, or protrusions, which predispose leg to trauma	—	1	3	1	2	—	7
5	Deep skin folds whose base cannot be seen when patient moves the leg	1	—	—	—	—	—	1

a Stage of lymphoedema is taken from Dreyer et al. [22], who identify up to 7 stages. Stages 6 and 7 are more extreme than stage 5 and were not encountered in patients in this study.

Thirty of the 60 participants with LF were selected from three villages: Polhena, Wagama, and Madihe in Matara District. A survey in 2003 [20] identified 117 cases of lymphoedema of varying stages, and six more were identified subsequently. Of the total 123, 107 consented to take part in a lymphoedema management experiment. A sample of 30 was selected from the 107 cases for qualitative interviews, to include a balance of women and men, of poor/nonpoor status, and of lymphoedema stage. Poverty status was identified for the initial sample selection from the questions on occupation in the epidemiological survey, with participants in informal labour occupations categorised as poor (the subsequent in-depth interviews provided detailed information on income to define participants' income status more directly). The main stages of advance of lymphoedema were graded according to the classification of Dreyer et al. [22]. Six of the initial sample of 30 were willing to participate but were unable to complete the full interviews, and so were omitted from the analysis. Six replacements were selected from the 107 cases, all of whom completed the interviews.

A further 15 participants with LF were identified from a fourth village 10 km away from the villages in Matara District, Unawatuna (Galle District), chosen because it had no involvement in the lymphoedema management experiment. Key informants helped identify households containing people with LF. House visits were made to the named individuals and snowballing was then used to recruit further participants for interview. A total of 47 cases of lymphoedema were identified by this process, of which 15 were selected to provide a mix of sex, poor/nonpoor status, and lymphoedema stage. All agreed to participate in interviews.

A separate sample of 15 men with filarial hydrocoeles was selected from one village in Matara District where the Medical Officer of Health for the area considered hydrocoele to be prevalent. Local officials acted as key informants to help identify men with the condition. Recruitment by snowballing identified 42 cases, of whom 15 met the criteria and agreed to participate. Of the 15 men, three had undergone surgery for their condition, while 12 had not (Table 2).

**Table 2:** Sample Distribution—Men with Hydrocele by Income Category.

Status of Hydrocele	Household Income^a^			Total
	Low	Middle	High	
No surgery	8	4		12
After surgery	0	3		3
Total	8	7	-	15

a Income refers to current (at the time of interview) income.

### Qualitative interviews

A team of ten trained interviewers (four women and six men) supervised by a senior project officer conducted guided interviews with study participants in the local language, Sinhalese. Interviewers worked in single-sex pairs: one conducting the interview, the other recording the responses manually. To respect gender sensitivities of participants, the pairs of interviewers were assigned to interview participants of the same sex. Interview notes were transcribed and later translated into English for analysis. Interpretations of the data were fed back to, and refined with, the interviewers.

All the interviewers had at least 3 y of experience of conducting interviews according to the Affordability Ladder framework (see below). The discussions encouraged patients to “tell their story,” beginning with the first symptoms to the time of the interview, which covered periods ranging from 1 mo to nearly 30 y. Health-seeking behaviour, costs of access to treatment, and expenditure in the household at each stage were obtained through a historical profile of the disease and its consequences for the household economy. Although the participants recounted experiences that occurred over several years, most had vivid recall of the milestones in their illness because it had made significant marks in their lives. Each interview took 3–4 h, some being undertaken over 2 d. These would normally be considered exceptionally lengthy interviews, but this situation resulted not from the researchers' schedules but from the desire of the interviewees themselves to talk freely and at length about their experiences. Some commented that the interview itself provided a therapeutic release from long-pent-up emotions, as they had been socially isolated when their condition advanced.

### Definitions of terms relating to the Sri Lankan health system

#### Private practitioners

These are allopathic doctors practicing for a fee; sometimes referred to as “general practitioners”—very similar to family physicians.

#### Indigenous practitioners

These are private practitioners of Ayurvedic medicine. Many of them treat patients for a fee, charging for medicine that they prescribe and often provide to the patient at the consultation itself. Some are small-time general physicians, while others are specialists. Among them are many who have “inherited” knowledge and many who practice as private physicians.

#### Direct costs

These include physicians' fees (if private), drugs, transport (of the patient if outpatient, or if an inpatient, costs of visits by family members), diagnostic and other tests done in a private hospital or outsourced when facilities are unavailable in a government hospital

#### Indirect costs

Income foregone by the patient who must access a practitioner and/or a family member who needs to accompany the patient for treatment at a private or government hospital or private allopathic or Ayurvedic practitioner.

### Analysis

**Figure 1:**
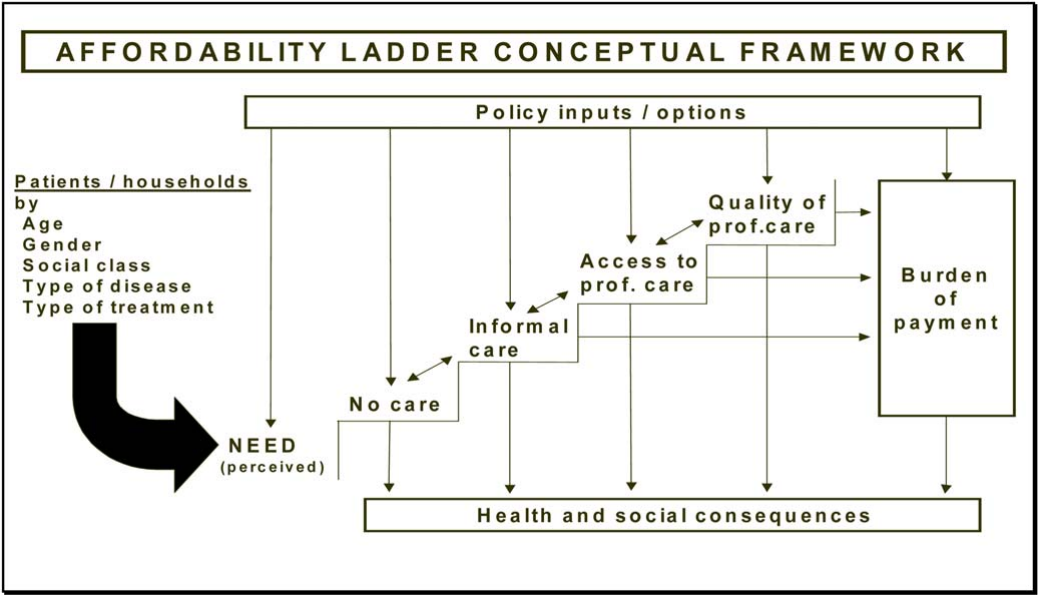
Affordability Ladder Conceptual Framework. Source: [24].

An adaptation of the affordability ladder framework [23] was employed to organise and analyse the data. Figure 1 [24] illustrates the basic conceptual framework. The starting point for the Affordability Ladder analysis on the left of the figure is a perceived or professionally defined health problem, a “need”; in this case, symptoms of LF. Perceived need and the consequences of that need may vary for different types of household depending on socioeconomic circumstances, and it is therefore important in the analysis to look at what happens to different groups in the population. Once the symptoms of LF are perceived by households, their experiences of seeking help for the condition may be very different in different types of households and are represented by the four main steps on the ladder: (1) No care; (2) informal care and/or self-care; (3) access to and utilisation of professional care; and (4) quality of professional care received. At each step of the ladder there are health and social consequences and a burden of payment as a result of the actions taken, as indicated by arrows. The policy environment also affects people's choices and actions in all these steps, as the arrows denote. These are not necessarily sequential steps: people may treat themselves with medicines or consult an informal provider, for example, at the same time as seeking professional help. The pattern of seeking care, however, may differ, again depending on socioeconomic differences.

In using a systematic approach to examine the many different aspects of the pathways from need to appropriate care, one important aim is to identify much more closely where and why the system is working well and where it is breaking down for different groups in the population [23].

**Figure 2:**
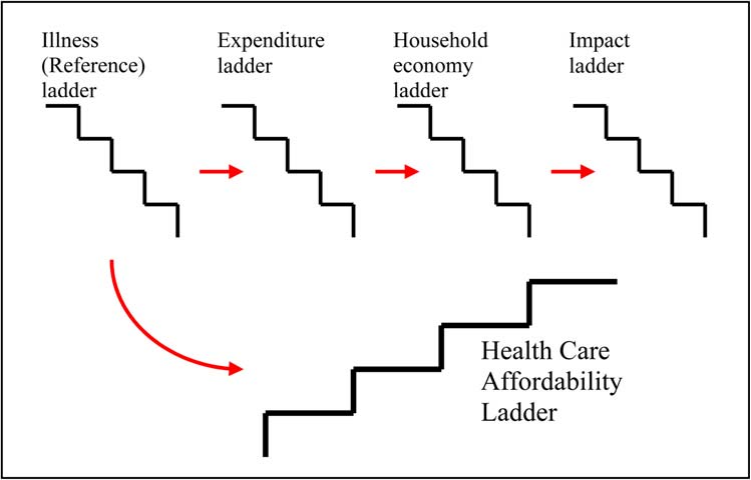
The Four Ladders Framework for Analysis of Qualitative Interview Data on Lymphatic Filariasis, Sri Lanka.

For the purposes of analysing the intricacies of participants' experiences from the qualitative data, we adapted this basic affordability ladder framework to incorporate four distinct ladders (Figure 2). The first, a *reference ladder*, documented the progression of the illness (historical profile of illness) as described above. A *treatment and expenditure ladder* recorded the direct and indirect costs incurred at each stage of the illness. The *household economy ladder* charted the changes in the household economy after a member of the household was identified as having LF and throughout the duration of the illness. Lastly, the *impact ladder* traced the economic and social consequences of the illness to the participant, the household and other family members. A process of constant cross-referencing each individual horizontally across ladders allowed interactions and the ordering of events and consequences to be identified. Recurring themes within and across the different types of household (low-, middle-, and high-income) were then identified by reviewing the entire dataset within the four-ladders framework. Emerging themes were noted, sorted, and grouped into main themes. Quotations and field notes describing interviewees' experiences with LF (presented in Boxes 1–4) are used to illustrate the main themes identified in the analysis.

From the income information obtained in the interviews, households were categorised as “high,” “middle,” or “low” income, judged against the level of incomes reported in the latest Consumer Finance Survey of the Central Bank [25]. “Low-income households” had income within the lowest three deciles range on the national scale, “high-income households” had income in the top three deciles range, and “middle-income households” had incomes that fell within the 4th to the 7th deciles range of the national scale. To minimise recall errors on expenditures, only costs incurred in the last 3 y, and costs for the most recent episode of inpatient care within the last 5 y, were used. Direct and indirect costs and costs as a proportion of the household income for participants in different types of household were calculated.

Names of study participants have been changed to protect their anonymity.

### Ethical review

The ethical approval for the study was obtained from the Research Ethics Committee of the Liverpool School of Tropical Medicine and the Ethics Committee of the University of Ruhuma, Galle, Sri Lanka. Verbal (oral) consent was given by the participants who were invited to participate; participants were reassured that they could withdraw from all or part of the interview at any time. The investigators judged, on the basis of their experience, that written consent was not obtainable because of the community-wide mistrust of signing any official forms and the level of literacy in the population. The ethics committees accepted this constraint. Studies conducted by the Marga Institute in similar settings have used the same approach, respecting the communities' concerns.

## Results

Participants' poverty and associated way of life severely limited their ability to prevent or cope effectively with the condition at all stages of the disease and its treatment. At the infection stage, for instance, poorer participants reported having to work for long hours in contact with stagnant water, with daily exposure to mosquito breeding places. Two common occupations for poor women—making coir yarn and weaving thatch—involved soaking materials in stagnant pits. Often the women had to stand chest-high in them for hours. These pits were generally sited adjacent to homes and were breeding places for *Culex quinquefasciatus*, the vector of *W. bancrofti.* The ability of patients to adopt preventive measures in the home was also severely limited. The poor could not afford the costs involved in avoiding exposure to mosquitoes, such as mosquito netting and repellent, and did not have the types of houses that would keep out mosquitoes.

When we traced participants' experiences in accessing and using the appropriate medical care for LF with the affordability ladder, multiple problems for households were revealed. Delayed diagnosis was common and had irreversible consequences. Both poor and nonpoor participants had experienced delays in diagnosis. The low-income households were more likely to report adopting home remedies for all types of illnesses, including lymphoedema. They tended to seek medical treatment only when the disease seriously affected their livelihoods, and then they tended to opt for indigenous treatment (which would not provide an accurate diagnosis), citing convenience, proximity, and cost as reasons.

Even low-income patients consulted private practitioners who charged a moderate fee but had no facilities to diagnose LF. These practitioners invariably treated only the immediate symptoms of fever and pain, and sometimes misdiagnosed the condition. One man, for example, had been treated for 7 y by an indigenous practitioner for the effects of “snake bite.” The lymphoedema of one pregnant woman went untreated for 9 mo because the symptom was judged to be due to the pregnancy.

Some participants reported that they chose to go to a private practitioner to avoid the social stigma brought about by exposure of their affected limbs at a government clinic, despite treatment being free at such clinics. The nonmedical costs of travel and the indirect costs in accessing a distant government hospital were also cited as reasons by low-income participants. The average reported delay from first symptoms to diagnosis for low-income participants was 3.5 y (range 2–7 y), while for middle- and high-income participants it was 2.2 mo (range 1–4 mo).

Once diagnosed, the ability of patients to follow prescribed drug treatment was severely constrained. Although a long course of the drug diethylcarbamazine (DEC) (currently 84 tablets, but previously up to 120 tablets [26]) was provided free by the Anti-Filariasis Campaign, side effects were reported by participants, who then interrupted or gave up the treatment. One low-income man said of the treatment, “In our daily routine and our struggle to find work each day, how can we think of tablets?” Another low-income woman commented: “Tablets can cause nausea and stomach cramps. Then we cannot go out and do our work.”

Travel costs and income foregone were deterrents for low-income participants to obtaining free drugs from the government hospital. An average direct cost of a visit to the hospital for outpatient care by a low-income participant was Rs 215 (US$2) which was the equivalent of 2 d of earnings for a low-income household. Inpatient care had an average direct cost of Rs 469 (US$4.50), which was equivalent to about 4 d of earnings for a low-income household. Corresponding indirect costs of income foregone because of attendance at health facilities amounted to 2 d of earnings for an outpatient visit and 15 d of earnings for an inpatient episode. Earned income in this population was not constant, but fluctuated from week to week and was unpredictable in poor households (see Box 1).

**Box 1. The Economic Loss and Social Stigma of Lymphoedema at Different Income Levels**

**Low-income family example**

**Raja** was 6 y old when he had the manifestations of LF with recurring episodes of fever, inflammation and pain. His mother could not always find the Rs 30 to hire a vehicle to take him to the hospital; hence he had to walk the 2 km to the hospital, dragging his painful leg. When such episodes recurred, rather than go through that ordeal he was given paracetamol (acetamenophen) to reduce the fever, which cost Rs 10 per card of 12 tablets. When he exposed the leg because he had to wear short trousers, he became the butt of jokes in school. He was instructed to visit the hospital clinic to obtain drugs every month and keep the leg bandaged with an elastic bandage, none of which his family could afford. He left school to become a labourer and so the hope the family had of improving their economic condition through education was lost. His mother developed asthma as a result of the stress caused by her son's condition. The father had to constantly interrupt his work to attend to the mother. Family income dropped to an uncertain one of Rs 1,500 (US$15) a month, with sporadic help from neighbours. The household was breaking down as they could barely afford a daily meal.

**High-income examples**

**Sriya:** “*I got this big leg when I was engaged to be married. When they heard it was filarial they backed out of the marriage. I was earning Rs 2,500 [US$25] a month from sewing but when the leg got worse the hospital doctor told me I should not pedal the machine. So I lost my income as well. When my parents died and my sister got married, only my brother and I lived in the house. My brother married and left the house, but my sister became widowed so came to live with me with her child. She had no money to buy bandage as instructed by the clinic. So I went to a house to cook. When they saw my leg they asked me not to come there anymore and found fault with me for hiding such a dirty illness from them. When I get fever I cannot walk to the hospital so I take paracetamol for two days and walk to the hospital when I feel less pain.*”

**Daya** was a woman from a middle-income household: “*I was only 20 years old when my leg swelled. I could not go to the government hospital which gave free treatment because people would see it and shun me and my family. Without treatment my leg got worse. I could not marry because of my disease. I lived with my sister and her husband. I was earning an income of Rs 3,000 [US$30]. When my leg got bad I sewed less garments and my income dropped to Rs 2,000 [US$20]. I could still continue to sew but my clients who saw my leg refrained from giving me orders for stitching. I lost my income and became dependent on my brother-in-law*.”

## 

More advanced stages of the disease were present among both poor and non-poor participants, but there were marked differences in the opportunities for participants from different types of household to manage their condition and ameliorate symptoms. Middle and high-income participants generally benefited from clean homes and facilities to maintain personal hygiene, they reported fewer episodes of fever and fewer injuries to the limbs, and they could afford bandages to reduce swelling of the limbs. Poorer participants lived in less-hygienic conditions and thus were more prone to infection, and they could not avoid frequent lesions and wounds because of the hazardous nature of their work. Several participants reported having wounds that turned into suppurating sores, but out of necessity they had continued to work with an infected limb.

More than half of the low-income participants reported that they could not afford the cost of attending the medical centre. The cost of frequent episodes of fever and swelling with pain was high for low-income participants. One low-income woman with lymphoedema had such episodes every 2 mo on average. She lost Rs 300 (US$3) income (equivalent to 6 d of female wages) from her thatch weaving, her husband lost the equivalent of 2 d of income (Rs 200 [US$2]) when he stayed at home to cook and look after the children and they spent about Rs 100 (US$1) during an episode on Panadol and herbal applications. A total loss of Rs 500–600 (US$5–6) per episode of illness had drained their income, putting them in debt.

The stigma associated with LF was a dominant theme in the accounts of most participants (see Box 1), which caused their condition to be hidden and contributed to delay in diagnosis with the subsequent advancement of the disease. For participants who had social standing in their village, social stigma tended to be a more important factor than costs in deterring them from seeking health care. Even high-income participants who reported no economic problems experienced mental health problems due to the stigma they suffered within the family.

Among the male participants, hydrocoele was a source of both physical suffering and intense social stigma. All 15 men recounted embarrassment and stigma associated with the hydrocoele, which had led them to hide their condition for years, until it was advanced and severely debilitating. Most low-income men earned their living from casual labour, mainly coconut picking, which involved climbing to great heights. They either had to give up the occupation, which caused loss of earnings, or continue to climb with the hydrocoele, thus greatly aggravating the condition. One man commented that his hydrocoele was as big as the coconuts he was picking, but he still had to continue working with it. (see Box 2).

**Box 2. The Impact of Hydrocoele in Men from Low-Income Households**

**Senas**' occupation of coconut picking had to be given up because his hydrocoele was so large and had become infected because he had injured it while climbing. His family was impoverished without his income. His son left school since he had to earn from casual labour. When Senas decided to have the surgery for the hydrocoele, his wife began working in domestic service cooking at a house close by. “*I did not consider it demeaning—what choice have when we are starving?*” She was given food from that house and a daily wage of Rs 50 [US$0.50]. This income, together with her sons' casual earnings, saw them through the 3–4 mo of convalescence after surgery. Senas was able to resume work, not in coconut picking, but in the lower-paid job of labourer. Their household income rose to Rs 4,000–5,000 [US$40–50] a month and the son returned to school.

Other men with hydrocoele could not afford a period off work for surgery and recovery. Men had jobs mainly in hard labour and coconut picking. They aggravated their condition by continuing with picking. “*I climbed with a hydrocoele as large as the coconuts I plucked.*” (**Indrasiri**, from a family who could not afford surgery, accepted a labourer's job, but even that was now becoming increasingly difficult to sustain.)

## 

The social and economic consequences for the whole household, not just the participant, spanned years. Loss of income because of the condition was reported by all households in the sample, across all income levels—it was not just confined to the poorest. The narratives of participants revealed reasonably well-off households, the members of which were gradually degraded into poverty by the condition over many years (Box 2). Equally, households that were already poor were pushed further toward destitution (Box 3). For some households in the sample, the presence of a member with LF had been a hindrance to family progress, rather than a cause of poverty, holding the family finances back when they could have achieved an improved standard of living. In one case, a family opted to deny the existence of the family member with lymphoedema, leaving him in a shabby room, given food, but unwashed and depressed, while other family members continued to make social and economic advancement (Box 4). A less extreme case of rejection by a high-income family (Box 4), illustrates the mental distress, as well as economic hardship, that was a consequence of lymphoedema.

**Box 3. Impoverishment in Low-Income Households**

**Bandu**, a low-income woman aged 32, developed lymphoedema as a young woman. She had a long period of self-medication, because that was her usual health-seeking behaviour. When she was diagnosed and began treatment the tablets provoked severe reactions of nausea and cramps, so she abandoned the treatment while her LF advanced. Her husband had to stay away from his work to nurse her during recurring attacks of fever and enlarged glands. Their income dropped from Rs 2,500 (US$25) to Rs 1,500 (US$15). She lost her income of Rs 500 (US$5) from coir work. Her daughter dropped out of school, her second daughter failed her public examination, and they became indebted to the grocer.

**Soma** had advanced lymphoedema, but did not access the Government hospital because she was ashamed to show her leg. She hid in her house for 15 y looking after her widowed daughter's children. She accepted money from her daughter to buy penicillin and paracetamol which she took daily to avoid fevers, but her unsightly leg showed no improvement. She was well on the way to become disabled and she was in despair: “*what will my poor daughter do if I cannot care for her children? She has only me*.”

**Box 4. Mental Illness Associated with Filariasis**

**Jamis** had stage 5 lymphoedema, which was infected and suppurating. He was a coconut picker and climbed trees until he could no longer. He had injured his limb by climbing. He was an embarrassment to his family. His children had dropped out of school because they were too poor to go to school. The sons became divers and acted as tourist guides and soon earned an adequate income. They built their house with tiled floors alongside the old shack. They were progressing up the social ladder; the one problem was their father with elephantiasis. He had been relegated to the shack where he lived in filth and dirt without income. He was given food and left to himself. “*I went with the tsunami but someone rescued me—why did I not die then?*” he said. He was depressive as a result. This was a family that rejected the undesirable element within it—their father—and moved ahead on a path out of poverty and into affluence, while Jamis waited for death, which he saw as a relief to his suffering.

**Ariya,** from a high-income household, had hidden her condition from her husband and his family. “*I could not take treatment since he would find out. The condition advanced and one day he found out my condition and began to shun me. I stopped going out anywhere specially where I might meet with his family. I am mentally broken down and do not know how long I can live like this, shunned and rejected and confined to this house.*”

## Discussion

While LF has been recognised for some time as a leading cause of disability globally, it has been relatively neglected by public health policy makers. Part of the reason for this neglect may be that the full extent of the disability associated with this disease is hidden and not recorded in standard assessments restricted to physical impairment. In this study, we have shown the extremely debilitating nature of LF over a long period of time when mental health, social, and economic consequences are taken into account using the affordability ladder framework. We have identified four areas in which the clinical manifestations of *W. bancrofti* infection had a major impact on the lives and livelihoods of patients and their families in Sri Lanka.

First, the condition and its diagnosis were severely affected by both stigma and costs. People with LF experienced the negative responses of others to their disfigured limbs or genitals, causing them to cover up the affected parts and, as the disease progressed, to hide themselves away from society in general. The social isolation from the stigma of the disease caused emotional distress, delay in diagnosis, and treatment, resulting in advancement of the disease beyond possible treatment.

Second, treatment services that were available—free—from Government clinics were avoided because the participants' condition would be publicly identifiable. Local private practitioners were favoured, where their condition could be more easily hidden. However, the consequence of this behaviour was that the patients received less effective, or even ineffective, treatment from private practitioners, compared with the interventions available through the government clinics.

Third, we found devastating economic and social consequences of the disease, for both patients and the household. The debilitating physical symptoms restricted the kind and quantity of work that participants could undertake, resulting in loss of earnings and impoverishment. Households were further impoverished by the costs incurred in using health services (even though the services themselves were free) and the cost of drugs, which had to be sustained over many years—leading to a medical poverty trap [27]. The impact of LF on productivity of the patients themselves can be considerable. In India, for example, a estimated US$842 million are lost to patients and households every year in treatment costs and reduced working time through acute and chronic disease caused by LF [7]. Other studies indicate that productivity loss in weavers can be as high as 27% [28], and male patients with chronic episodes of LF can lose an equivalent of 15% of their earning capacity in any one year [29]. A study of the costs of nonfilarial elephantiasis in Ethiopia provided similar estimates. Direct costs of podoconiosis (nonfilarial endemic elephantiasis of the lower leg) amounted to US$143 per patient per year with productivity lost per patient of 45% of working days, equivalent to monetary loss of US$63. The overall costs of this form of elephantiasis in one zone where the population is 1.5 million was estimated to be US$16 million per year [30].

For nearly all the participants in our sample, the incomes of other members of the household, in addition to the participant, were affected, either by having to forego employment to look after the patient or by making contributions to the health care costs. Several households in our sample had to withdraw children from school to help with work, which would perpetuate intergenerational poverty.

Fourth, the adverse social and economic consequences were socially patterned. While we found that households from all three income levels had suffered reductions in income, those who were already on low incomes were pushed into near destitution by LF, from which it was almost impossible to escape. Low-income households also had less opportunity to obtain effective treatment from distant clinics, coupled with living and working conditions that made hygiene and compliance with treatment regimes more difficult. They were also less protected from stigma.

These findings have significant policy implications. In Sri Lanka the prevalence of filarial elephantiasis in the population of three villages in Matara district has been estimated to be 3% and the prevalence of hydrocoele to be 6.2% [21]. The villages are typical of endemic areas in terms of socioeconomic mix and occupations. Scaling up the estimates to the whole 10 million population of the endemic provinces gives an expected 300,000 cases of elephantiasis among both women and men, and around 300,000 men with filarial hydrocoele from a male population of approximately 4.8 million. Every afflicted person lives in a household with another four individuals on average, all of whom may potentially suffer social and economic consequences as a result of having a family member with this condition. Even if the LF elimination programme is successful in arresting transmission of the disease so that there are no new cases, hundreds of thousands of people in Sri Lanka will continue to suffer clinical manifestations of the disease and will remain trapped in poverty. The affected households will need help and support for many years despite transmission having been arrested.

Donors and the national government, who have to date understandably focussed on the single-dose annual preventive strategy where DEC and albendazole (400 mg chewable tablet donated by GlaxoSmithKline) have been given annually since 2001, need to re-evaluate how this neglected group can be served more effectively. This will first require a comprehensive survey to ascertain the number of people with elephantiasis and hydrocoele in the endemic districts. This survey needs to be followed by a reassessment of post-Mass Drug Distribution strategies, expansion of the lymphoedema treatment programmes to maximise coverage for those that remain symptomatic, and an aggressive approach to the provision of surgical care for male patients with hydrocoele.

Direct financial support to afflicted families could be provided under the Sri Lankan Samurdhi poverty alleviation scheme of allowances for stipulated poor households. This approach could then take account of the context and barriers to effective treatment when designing and scaling up the post-Mass Drug Distribution implementation programmes. Supporting the evaluation of clinical interventions for the amelioration of lymphoedema will also be important, as the interventions currently being piloted in Sri Lanka and elsewhere need to recognise the social and economic context and constraints on the lives of participants, and how these differ by level of poverty.

The inclusiveness and the caring quality of a health strategy for any given disease has to be judged by its capacity to reach out to the most vulnerable groups affected. The present study demonstrates one of the dilemmas that can arise in a strategy for the control and prevention of a disease leading to chronic conditions of ill health such as LF. The strategy itself, as in the case of Sri Lanka, can achieve its main objectives of prevention and elimination of the disease through large-scale interventions that reach the great majority of the population exposed to it. However, a highly vulnerable category of patients in advanced stages of the disease tends to have low visibility, becoming marginalized and forgotten. Special measures are needed to identify, reach and care for them. As the Global Filariasis Elimination Programme reports successes in arresting transmission, those with the condition should not be neglected but be specially targeted for the support the condition requires. Such support could be promoted by specific poverty reduction policies, which would be entirely appropriate given the evidence presented in this paper of the impact of the disease on poor communities, and particularly at the household level.
